# Nondisclosure prosecutions and population health outcomes: examining HIV testing, HIV diagnoses, and the attitudes of men who have sex with men following nondisclosure prosecution media releases in Ottawa, Canada

**DOI:** 10.1186/1471-2458-13-94

**Published:** 2013-02-01

**Authors:** Patrick O’Byrne, Jacqueline Willmore, Alyssa Bryan, Dara S Friedman, Andrew Hendriks, Cynthia Horvath, Dominique Massenat, Christiane Bouchard, Robert S Remis, Vera Etches

**Affiliations:** 1 Ottawa Public Health, Ottawa, Canada; 2 Faculty of Health Sciences, School of Nursing, University of Ottawa, 451 Smyth Road, Ottawa, ON, K1H 8M5, Canada; 3 University of Toronto, Toronto, Canada

**Keywords:** Canada, Criminal law, Disclosure, HIV, Population health, Testing, Qualitative, Quantitative

## Abstract

**Background:**

During the past decade, the intersection of HIV and criminal law has become increasingly discussed. The majority of studies to date have approached this topic from a sociological or legal perspective. As a result, the potential effect of nondisclosure prosecutions on population health and HIV prevention work remains mostly unknown.

**Methods:**

A descriptive quantitative-qualitative study was undertaken to examine HIV testing, HIV diagnoses, and the attitudes of men who have sex with men following regional media releases about a local nondisclosure prosecution. As part of this study, first, we reviewed the trends in HIV testing and HIV diagnoses from 2008 through 2011 in Ottawa, Canada. Second, we explored the attitudes and beliefs of local MSM about HIV, HIV prevention, HIV serostatus disclosure, nondisclosure prosecutions, and public health.

**Results:**

Quantitatively, the findings of this study revealed that, in comparison to the period preceding the media releases about a local nondisclosure prosecution, HIV testing and HIV diagnoses among men who have sex with men did not significantly change after the media releases of interest. Qualitatively, a subgroup of 27 men who have sex with men (12 HIV-positive, 15 HIV-negative) noted their beliefs that the local public health department openly shares information about people living with HIV with the police. Moreover, some HIV-positive participants stated that this perceived association between the local public health department and police services caused them to not access public health department services, notwithstanding their desires to seek assistance in maintaining safer sexual practices.

**Conclusions:**

Nondisclosure prosecutions likely undermine HIV prevention efforts.

## Background

On May 6, 2010, the Ottawa Police issued a media release about a person living with HIV/AIDS (PHA) who they alleged did not disclose his HIV-positive serostatus prior to engaging in activities that posed a “significant risk” for HIV transmission. This release, which included the accused’s photograph, name, and sexual orientation, was widely covered by local and national media. Media attention included electronic publications, radio discussions, and print media. From May 6 to August 23, there were more than 45 news articles on the case. To the best of our knowledge, these media releases constituted the first high-profile nondisclosure prosecution in Ottawa where such detailed information was provided to the public.

Some authors have voiced concern that this use of the criminal law (henceforth referred to as nondisclosure prosecutions) may negatively affect HIV prevention [1,2]. Others, such as the Supreme Court of Canada in its 1998 *R. v. Cuerrier* ruling, [3] suggested that such prosecutions would have favourable public health benefits and would not affect HIV testing:

The criminal law has a role to play both in deterring those infected with HIV from putting the lives of others at risk and in protecting the public from irresponsible individuals who refuse to comply with public health orders to abstain from high-risk activities (p52).

It is unlikely that individuals would be deterred from seeking testing because of the possibility of criminal sanctions arising later (p61).

In 1998, the Supreme Court of Canada thus articulated beliefs about the relationship between nondisclosure prosecutions and HIV testing.^a^ They did so, however, without evidence. Contributing to this problem is that, to date, only a few researchers have examined the relationship(s) between nondisclosure prosecutions and HIV prevention [4-8].

In light of this knowledge gap and the local situation, we undertook a descriptive quantitative-qualitative project that focused on men who have sex with men (MSM) because, first, this was the focus of the media releases, and, second, over half of the HIV cases diagnosed in Ottawa in 2010 were among MSM. The quantitative component of this study examined the number of HIV tests and HIV diagnoses during a four-year period; the goal was to assess if there was a change in HIV testing or HIV diagnoses following the media releases. The qualitative component of this project explored the self-reported attitudes and behaviours of 27 MSM regarding HIV-serostatus disclosure, nondisclosure prosecutions, and public health; 12 of these participants reported being HIV-positive, and 15 HIV-negative.

This research differs from previous work because the present study examined population health outcomes (e.g., HIV testing and HIV diagnoses), not public health practice (e.g., the ability of public health officials to undertake HIV prevention). Notwithstanding this novel focus, one must interpret our data in light of its limitations. First, as Dodds [9] aptly suggested, “assessment of the impact of prosecutions at a population level … cannot be achieved … by examining overall HIV testing rates, as [sic] the testing patterns of populations are influenced by a wide range of factors that can pull in different directions at the same time” (p136). Second, we are unsure if our qualitative findings could be replicated with different research samples. We do not know if the themes we identified emerged simply due to the temporal proximity of local nondisclosure prosecution media releases.

### Population health HIV prevention

**Figure 1:**
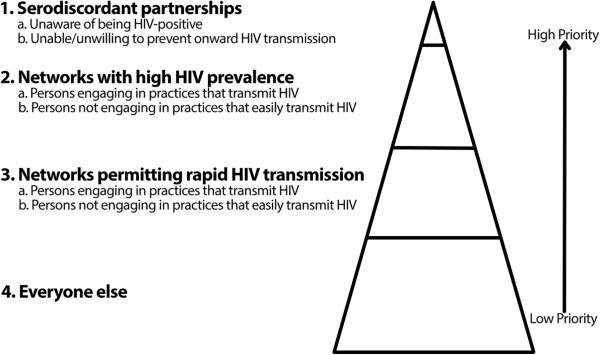
**A population health HIV prevention framework.** (Adapted from Aral et al.[10]).

Because HIV transmission requires contact between a person who is HIV-positive and another who is not (i.e., serodiscordant partners), HIV prevention should focus on (a) persons, both HIV-positive and HIV-negative, who engage in practices that transmit HIV with serodiscordant partners, and (b) HIV-negative persons who engage in practices that transmit HIV, and who belong to the groups most affected by HIV [10,11]. See Figure 1.

Regarding the first of the foregoing groups, Marks and colleagues [12,13] emphasized an important point: In the US, people unaware of being HIV-positive are probably involved in 54-70% of HIV transmission. The foundation of Marks and colleagues’ [12,13] conclusion was the literature which highlights how most persons, when diagnosed with HIV, become less likely to transmit HIV because they adopt safer sex practices, diminish their HIV viral loads with, medications and have progressed through the acute HIV infection period [12-16]. If Marks and colleagues’ [12] estimates were similar for Canada, wherein, as of 2011, approximately 25% of PHAs were unaware of their serostatus, [17] then enhanced serostatus awareness among persons who do not know they are HIV-positive could decrease HIV transmission due to the aforementioned behaviour changes, and as a result of improved access to treatment and subsequent viral load suppression [12-16]^b^.

Despite most HIV transmission likely involving persons unaware they are HIV-positive, research indicates that some PHAs aware of their serostatus continue to engage in practices that transmit HIV [18]. According to the Ontario Advisory Committee on HIV/AIDS, [18] these persons are either “unwilling or unable to take precautions and, therefore, put others at risk” (p.2). While these individuals are likely implicated in numerically fewer HIV transmissions than individuals who are unaware of being HIV-positive, they nevertheless constitute an important target group for prevention. In such cases, the Ontario Advisory Committee on HIV/AIDS [18] recommends education, support programs, and public health involvement because these approaches address the unique life-situations PHAs face.

## Methods

This study involved quantitative and qualitative data collection components. Ethics approval was obtained from Ottawa Public Health’s Research Ethics Board.

### Quantitative component

The goal of the quantitative component of this study was to assess whether there were any short-term changes in HIV testing or HIV diagnoses among MSM in the local region in the four months following the media release of interest. This aspect of our study thus examined HIV testing among persons who belong to *group two* in our population health HIV prevention framework; i.e., persons who belong to sexual network with high HIV incidence and prevalence. See Figure 1. Examination of HIV diagnoses, by comparison, measured HIV testing uptake within the subpopulation of MSM who are unaware of being HIV-positive; recent estimates identify that approximately 20% of MSM in Canada belong to this group [17]. Based on the nature of this research (i.e., a descriptive analysis of population-level data about the number of MSM who self-select to undergo HIV testing and the number who test positive for HIV), definitive conclusions about the relationships between changes in HIV testing or HIV diagnoses after the media releases would be inappropriate.

#### Study design

We examined the monthly number of HIV tests and HIV diagnoses in the Ottawa Public Health region from 2008 through 2011. To do this, we created two time periods of four months both preceding and following the initial media release (May 6th) for each year: January through April (“pre”) and May through August (“post”). We obtained data on HIV testing and HIV diagnoses in the local jurisdiction from the Public Health Laboratory – Public Health Ontario. Essentially all diagnostic HIV testing in Ontario is carried out at this laboratory.

For testing and diagnosis data, we allocated HIV tests associated with individuals with unknown sex or region of residence based on the distribution among tests of individuals with known sex or residence; this information was available for over 95% of HIV tests and diagnoses [19]. We categorized HIV tests and diagnoses into exposure categories (e.g., MSM) based on risk factors indicated on the HIV laboratory requisition. We used information from the Laboratory Enhancement Program (LEP) to (a) allocate HIV tests without risk factor information (NIR) and (b) to re-allocate HIV tests in the low-risk heterosexual exposure category [19]. We carried out these re-allocations because surveillance category data were available from laboratory requisitions for only half of the HIV tests, and because results from the LEP indicate that some individuals are mistakenly reported as belonging to the low-risk heterosexual group [19]. In this study, we refer to the number including the allocated and re-allocated categorizations as “adjusted” exposure categories.

#### Analysis

We examined the total number of HIV tests and HIV diagnoses among MSM by month using monthly totals and three-point moving averages of unadjusted and adjusted cases. We selected a p-value of 0.05 before beginning analysis to determine statistical significance, and used Stata/SE v.12.0 for analyses. We used analysis of variance (ANOVA) to determine if there was a significant change in the mean number of monthly tests between time periods, and we undertook paired, two-sided t-tests to compare the mean number of tests in the pre and post periods within years, and to compare the mean number of tests in the post period of one year to the post period of another year. The two-sided Fisher’s exact test was used to test for significant differences between the proportion of positive and negative HIV diagnoses among MSM over time, using the same comparisons described above. Lastly, we only considered first-time positive HIV tests as diagnoses in our analysis.

### Qualitative component

The qualitative component of our study gathered in-depth information from local gay, bisexual, and other MSM about HIV-serostatus disclosure, nondisclosure prosecutions, and public health. These interviews were 60-90 minutes and semi-structured. New interviews continued until data saturation, which occurred at the point that no new findings were identified in the data (described below). Participants received a CAD $20 honorarium.

#### Inclusion/exclusion criteria and recruitment

The sample inclusion criteria from the research participants were: MSM; lived in the local region; spoke English or French; aware of the recent nondisclosure prosecution media releases. Individuals not meeting these criteria were excluded. Recruitment involved increasing awareness about the project with community agencies, distribution of posters in venues frequented by MSM, and snowball sampling. As part of the last strategy, participants were asked, without obligation, to refer others who might participate.

#### Data analysis

The data were assayed using a six-step constant comparative thematic approach [20]. First, the research team read and discussed the interview transcripts multiple times. This enhanced understanding of the participants’ data. Second, after more detailed reviews of the transcripts, we generated codes, which described the participants’ statements. Codes were tracked in an Excel file to determine data saturation. Specifically, the codes were listed on the vertical axis and the interviewees were listed on the horizontal axis; when multiple interviews failed to expand the code list, saturation was established. Third, after the data were saturated, we combined codes to form themes, and named these themes. None of these titles existed in the transcripts; rather, they described the common element(s) that linked the categorized codes. Fourth, we evaluated the themes to ensure the codes were appropriately aggregated. To facilitate this process, a secondary review of the interview text occurred to ensure that, as we worked with the codes and not the raw data, the codes did not deviate from the participants’ meaning. No deviations were found. Furthermore, we also sought out codes that did not fit the thematic structure we had produced. This helped ensure that our themes were robust, distinct, and internally cogent. Fifth, we defined each theme. Here, we established the nature and scope of each theme, and detailed its relationships to the other themes. Sixth, we produced an overarching narrative to describe the data.

## Results

### Quantitative results

**Figure 2:**
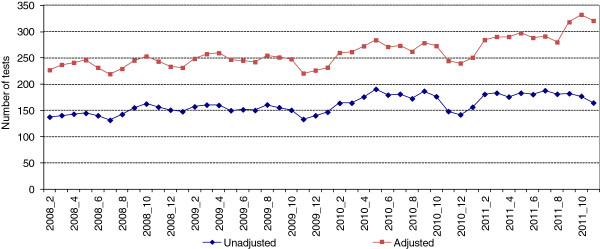
**Total HIV tests among MSM (unadjusted and adjusted**^**a**^**) by month, Ottawa, 2008- 2011 (3-point moving average).**

The number of monthly HIV tests performed among MSM increased from January 2008 to December 2011 (Figure 2). The decrease in November and December 2010 was primarily due to the closing of one of the main testing locations, which performed 17% of male HIV tests in Ottawa in 2009, throughout the last two weeks of November and all of December. In addition, a peak in late 2011 in the adjusted number of MSM tests was likely an overestimate caused by an increase in the number of no information reported (NIR) tests in October through December 2011. This increase is likely related to an investigation into a community infection control lapse that used a laboratory requisition for HIV testing that did not contain any risk factor information.

The analysis we undertook to assess changes in testing across the eight time periods showed a significant between-group difference when using both unadjusted and adjusted number of tests (F_7,31_ = 7.46, p < .01, and F_7,31_ = 8.44, p < 0.01, respectively). This confirmed that the observable increase in HIV testing from January 2008 to December 2011 was statistically significant. There was no significant change in the mean number of unadjusted or adjusted HIV tests in the four months following the media release compared to the four months preceding it (Table 1). An increase in the mean number of unadjusted and adjusted HIV tests from May through August 2010 compared to May through August 2009 was not statistically significant either (p = 0.072 and 0.094, respectively) (Table 2).

**Table 1:** Paired t-tests of mean number of monthly HIV tests among MSM (unadjusted and adjusted) in Ottawa within years, 2008-2011

**Pre-post periods**	**Unadjusted MSM tests**				**Adjusted**^**a**^**MSM tests**			
	**Mean**	**Difference**	**% change**	**p-value**	**Mean**	**Difference**	**% change**	**p-value**
Jan-Apr ′08	140.5				236.3			
May-Aug ′08	137.5	−3.0	−2.1%	0.71	227.3	−9.0	−3.8%	0.52
Jan-Apr ′09	159.5				253.5			
May-Aug ′09	147.8	−11.8	−7.4%	0.27	241.0	−12.5	−4.9%	0.34
Jan-Apr ′10	167.3				263.3			
May-Aug ′10	180.0	12.8	7.6%	0.48	271.8	8.5	3.2%	0.66
Jan-Apr ′11	179.5				285.8			
May-Aug ′11	184.0	4.5	2.5%	0.66	291.0	5.3	1.8%	0.68

Source: Public Health Laboratory. Public Health Ontario. Extracted April 23, 2012.

^a^Adjusted for unknown region, unknown sex and unknown exposure category using LEP.

**Table 2:** Paired t-tests of mean number of monthly HIV tests among MSM (unadjusted and adjusted) in Ottawa between years, 2008-2011

**Annual comparisons**	**Unadjusted MSM tests**				**Adjusted**^**a**^**MSM tests**			
	**Mean**	**Difference**	**% change**	**p-value**	**Mean**	**Difference**	**% change**	**p-value**
May-Aug ′08	137.5				227.3			
		10.3	7.5%	0.31		13.8	6.1%	0.21
May-Aug ′09	147.8				241.0			
		32.3	21.8%	0.072		30.8	12.8%	0.094
May-Aug ′10	180.0				271.8			
		4.0	2.2%	0.822		19.3	7.1%	0.34
May-Aug ′11	184.0				291.0			

Source: Public Health Laboratory. Public Health Ontario. Extracted April 23, 2012.

^a^Adjusted for unknown region, unknown sex and unknown exposure category using LEP.

**Figure 3:**
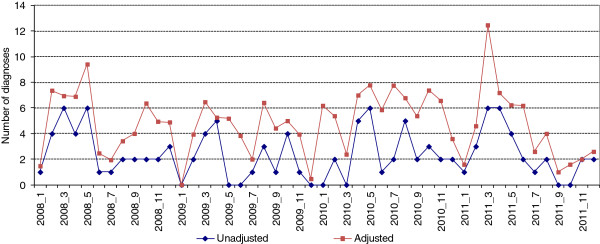
**HIV-positive diagnoses among MSM (unadjusted and adjusted**^**a**^**) by month, Ottawa, 2008-11.**

Figure 3 shows no apparent trend in the unadjusted and adjusted number of HIV-positive diagnoses among MSM from January 2008 through December 2011. There was no significant change in the proportion of either unadjusted or adjusted positive HIV test results in the four months following the media release compared to the four months preceding it (Table 3). Using unadjusted values, we noted a higher proportion of positive HIV test results in the four months post-media release in 2010 (14 positive, 706 negative, 1.9% positivity), compared to the same post-media release period in 2009 (4 positive, 587 negative, 0.7% positivity). The unadjusted figures, illustrated in Figure 3, also highlighted a marked drop in HIV diagnoses among MSM in Ottawa in the month immediately following the media releases in question. Our comparison of these numbers, however, did not yield statistical significance (p = 0.057) (Table 4). We found no other differences between the time periods of interest that approached *p* = 0.05 (Table 4).

**Table 3:** Fisher’s exact tests of proportion of positive HIV test results among MSM (unadjusted and adjusted) in Ottawa within years, 2008-2011

**Pre-Post periods**	**Unadjusted MSM tests**				**Adjusted**^**a**^**MSM tests**			
	**Positive tests**	**Negative tests**	**% positivity**	**p-value**	**Positive tests**	**Negative tests**	**% positivity**	**p-value**
Jan-Apr ′08	15	547	2.7%		22.7	923.2	2.4%	
May-Aug ′08	10	540	1.8%	0.42	17.3	891.9	1.9%	0.43
Jan-Apr ′09	11	627	1.7%		15.7	997.5	1.5%	
May-Aug ′09	4	587	0.7%	0.12	17.4	946.0	1.8%	0.86
Jan-Apr ′10	7	662	1.0%		21.0	1032.0	2.0%	
May-Aug ′10	14	706	1.9%	0.19	28.2	1059.4	2.6%	0.39
Jan-Apr ′11	16	702	2.2%		25.9	1115.7	2.3%	
May-Aug ′11	9	727	1.2%	0.16	19.0	1144.5	1.6%	0.29

^a^Adjusted for unknown region, unknown sex and unknown exposure category using LEP.

Source: Public Health Laboratory. Public Health Ontario. Extracted April 23, 2012.

**Table 4:** Paired t-tests of proportion of positive HIV test results among MSM (unadjusted and adjusted) in Ottawa between years, 2008-2011

	**Unadjusted MSM tests**				**Adjusted**^**a**^**MSM tests**			
**Annual comparisons**	**Positive tests**	**Negative tests**	**% positivity**	**p-value**	**Positive tests**	**Negative tests**	**% positivity**	**p-value**
May-Aug ′08	10	540	1.8%		17	892	1.9%	
				0.11				0.86
May-Aug ′09	4	587	0.7%		17	946	1.8%	
				0.057				0.23
May-Aug ′10	14	706	1.9%		28	1059	2.6%	
				0.30				0.14
May-Aug ′11	9	727	1.2%		19	1145	1.6%	

Source: Public Health Laboratory. Public Health Ontario. Extracted April 23, 2012.

^a^Adjusted for unknown region, unknown sex and unknown exposure category using LEP.

### Qualitative data

#### Demographic information

A total of 27 participants were interviewed (12 HIV-positive, 15 HIV-negative); 23 provided demographic information (Table 5.)

**Table 5:** Demographic characteristics

**Age:**	**Total:**	**HIV -**	**HIV +**
**19-30**	11 (48%)	9 (82%)	2 (18%)
**31-40**	7 (30%)	1 (14%)	6 (86%)
**41-50**	3 (13%)	1 (33%)	2 (67%)
**51-60**	2 (9%)	1 (50%)	1 (50%)
**Salary:**	**Total:**	**HIV -**	**HIV +**
**<20,000**	12 (52%)	5 (42%)	7 (58%)
**21,000 - 40,000**	3 (13%)	2 (67%)	1 (33%)
**41,000 - 60,000**	3 (13%)	2 (67%)	1 (33%)
**61,000 - 80,000**	4 (17.5%)	2 (50%)	2 (50%)
**81,000 - 100,000**	0	0	0
**>100,000**	1 (4.5%)	1 (100%)	0
**Education:**	**Total:**	**HIV -**	**HIV +**
**Elementary**	3 (13%)	0	3 (100%)
**High School**	3 (13%)	1 (33%)	2 (67%)
**College**	4 (17.5%)	2 (50%)	2 (50%)
**Bachelor’s degree**	10 (43.5%)	7 (70%)	3 (30%)
**Graduate**	3 (13%)	2 (67%)	1 (33%)
**Ethnicity:**	**Total:**	**HIV -**	**HIV +**
**Caucasian**	20 (87%)	10 (50%)	10 (50%)
**Aboriginal**	1 (4.3%)	0	1 (100%)
**Black**	1 (4.3%)	1 (100%)	0
**Other**	1 (4.3%)	1 (100%)	0

#### Interview findings

Two noteworthy themes emerged from the qualitative data: (1) perceptions of PHAs; and (2) linking public health and police services. Please note the names reported below are pseudonyms.

#### Theme 1: Perceptions of PHAs

The first finding of the qualitative component of our study related to the two main ways the participants described PHAs. First, they reported their beliefs that, within society, there is a perception that PHAs acquired HIV due to promiscuity. Two participants stated:

I think that society looks at people with HIV as often being people who were promiscuous and deserving what they got (Eric, HIV-, 19-30 years old).

[When my mum] told my family [I was HIV-positive], … she made it sound like I went out and had sex with every guy and got HIV. (Quincy, HIV+, 31-40 years old).

Above, Eric and Quincy described their perceptions about how some people associate HIV transmission with promiscuity. In its full scope, however, this first theme moved beyond sexuality and described how some of our HIV-negative participants also believed that PHAs are inherent or latent criminals. Martin described this belief as follows: “A man becomes a weapon because he can kill with HIV” (HIV-, age unknown). Victor, meanwhile, described unprotected sex by PHAs as murder: “As far as I’m concerned, you just killed the guy; you’re a murderer” (HIV-, 41-50 years old). In these two quotations, perceptions about HIV do not relate to the health or sexuality-related facets of PHAs, but rather, were law-focused discussions about PHAs’ *latent* criminal tendencies. Note in these quotations the inherent association between HIV, people living with HIV, and the criminal law. Cedric (quoted below) exemplified this association between HIV and the criminal law when, in the next two quotations, he described PHAs as “criminals” who “should go to jail” if they have “unprotected sex”. Cedric stated:

I think anyone who knows they have HIV and has unprotected sex should go to jail because that is a crime. You’re infecting someone with a disease (Cedric, HIV-, 19-30 years old).

That [nondisclosure prosecutions] could save someone from getting HIV from someone who had sex with a criminal (Cedric, HIV-, 19-30 years old).

Underpinning Cedric’s statements is an unannounced association between HIV and the criminal law. Regardless of intent or actual HIV transmission outcomes, having "unprotected sex" should mean "jail because that [behaviour] is a crime. An HIV infection, accordingly, corresponds with presumptions about latent criminality: Many of our HIV-negative participants believed that PHAs possess such potential criminal tendencies because of their serostatus. Further exemplifying this finding, Eric and Seamus described how an individual who becomes HIV-positive acquires not only a viral infection, but also a label that purports criminal capabilities and tendencies:

If this is the first time you find out you’re HIV-positive, all of a sudden it blow[s] up and we’re involving everyone. Everyone in a uniform is now involved and you have no time to be treated as a person who had been dealt pretty significant news. (A) How stigmatizing is that for you as an individual? All of a sudden, I have no rights and I’m a criminal, potentially. (B) People are not concerned about my mental health; they’re concerned about tracking this down in other people. (C) I have to disclose everything right now, but I haven’t straightened anything in my head. I haven’t become comfortable or been treated like a person (Eric, HIV-, 19-30 years old).

Seamus supported Eric’s sentiments:

For HIV-positive people, psychologically, we’ve already labelled [them] as criminals … That’s damaging to HIV-positive people and the negative people because it’s a risk of me being labelled a criminal automatically (Seamus, HIV-, 19-30 years old).

Above, Eric and Seamus described their perceptions that we have “labelled [PHAs] as criminals” not because of any illicit action or activity, but because of their serostatus. The current focus, according to Eric, is on “tracking down [HIV] in other people” and “[serostatus] disclos[ure]”, not on “treating [a PHA] like a person”. The primary concern, in other words, is protecting society from the alleged harm of potential criminals. One main finding of our results, therefore, was that many HIV-negative participants linked an HIV-positive serostatus with the criminal law.

Furthermore, and of particular interest, Seamus reported that the criminal law is not only “damaging” for PHAs, but also for people who believe they are HIV-negative, particularly if these individuals fear they might be HIV-positive. This latter point relates to concerns that knowing one is HIV-positive corresponds with additional duties of serostatus disclosure and the potential ramifications of serostatus nondisclosure. Seamus stated:

If I don’t have it [HIV], and I truly don’t know that I may have HIV … well then how can I be charged of a crime if I give somebody HIV? … That would scare people from getting tested (Seamus, HIV-, 19-30 years old).

Here, Seamus suggested that nondisclosure prosecutions could undermine HIV testing rates. Eric supported this finding:

I think it [the local nondisclosure prosecution media release] changed testing behaviour; I don’t think [however] that it changed risk-taking behaviour because everyone I know seems to be a little bareback slut (Eric, HIV-, 19-30 years old).

Eric’s statement added an important detail. While he believed HIV testing may have changed as a result of nondisclosure prosecutions, he had not seen any decreases in unprotected sex. This statement, in combination with those from above, illustrated the two aspects of our participants’ descriptions about PHAs. On the one hand, the participants outright stated that the broader social perception about PHAs was that HIV infection was the outcome of promiscuity. On the other hand, more in-depth analysis of our HIV-negative participants’ statements about PHAs revealed that their beliefs about HIV-positive persons were supported by what these participants believed was a natural association between HIV and the criminal law; we labelled this relationship ‘the latent criminality associated with being HIV-positive’. While some participants speculated that this latent criminality could deter persons from undergoing HIV testing, the main aspect of this qualitative finding was that our participants linked HIV with the criminal law as if this association were inherent.

#### Theme 2: Linking public health and police services

Our second theme described the participants’ beliefs about how public health and police services are linked in the local context. This finding emerged because, when asked about how they perceived local public health practices since the nondisclosure prosecution media releases, the participants indiscriminately referred to the public health department and the local police force. In other words, our participants did not see a clear boundary between the local public health department and the local police service. The following quotations exemplify this perception:

**Has it [the media release] changed your perceptions of public health?** … My answer would be no. I haven’t changed my perception of public health. It’s reinforced that [the health department], along with the police, were aware of this. … I think [the health department] involvement was to identify him and to release this information to the police (Henry, HIV-, 19-30 years old).

I think the main intention was over the line, but not the objective: To protect the public. So, I support criminalization, prosecution, the manner in which public health officials want to inform people (George, HIV-, 41-50 years old).

Above, Henry described the perception that the local health department and police services transfer and release information between them. George reinforced this association when he described “criminalization”, “prosecution”, and “public health officials” each as an interdependent component of the “public protection” apparatus. Further reinforcing this finding were George’s associations of “criminalization, prosecution” and “the manners” used by “public health officials”. Here, George described the media release in question as the method by which public health officials informed people about HIV, and nondisclosure prosecutions as a method for public health officials to ensure HIV prevention. It is important to note, however, that the police, not the local public health department, released the media releases of interest.

When we asked PHAs about the local public health department, similar responses emerged. For example, Jacob indicated that he too believed that the local public health department and the local police force were linked. In fact, Jacob believed that the criminal prosecution was a public health activity (please note the italicized text below) – a sentiment that he followed with a statement about how he wished he had undergone anonymous HIV testing after learning about “the criminal charges possibility”.^c^ In other words, Jacob wished that the local public health department did not know his HIV-positive serostatus:

From what I had read and heard it was mostly you’d get a notice under some section of public health [law] ordering you to disclose all the time. *And then if that was violated they [i.e., public health officials] would move towards criminal charges and whatnot.* But they would normally start out with a… I can’t remember what they used to call it, a section something or other. But that was my impression of what it was, so I didn’t even realize … the criminal charges possibility. Looking back I’m kind of like, oh wow, maybe I should have let the Public Health Nurse do this [HIV test] anonymously (Jacob, HIV+, 19-30 years old). (*Emphasis added*)

Similar to Jacob, Theodore (quoted below) reinforced the finding that the participants linked the local public health department and the local police force. In this next quotation, Theodore noted that the local public health department “tried to remove themselves” from their attachment—and thus natural or inherent linkage—to the police. Theodore stated:

[The public health department] tried to play both sides of the fence. They tried to remove themselves from the police, being like ‘it was the police that put up this press issue.’ … But I don’t feel safe going to public health with my issues. I’m not sure if I were to talk openly about my experiences, or what’s going on, or the risky sex I had, if it could be used against me. I always feel that they’re building records … , and all of this can be used against me. I don’t think that they’re a partner [of PHAs]; I think that they’re more against us than for us (Theodore, HIV+, 19-30 years old).

According to Theodore, regional nondisclosure prosecution media releases led him to believe that he could not trust the local public health department because they were “building records [that] … can be used against [him]”. These beliefs made him not “feel safe going to public health”, for example, to discuss “the risky sex [he] had”. This assertion encapsulated a common sentiment expressed by our participants: that public health and police services are inextricable, and, therefore, that public health workers are not a safe option for discussing previous sexual practices if these practices were illegal. Theodore’s statement also corresponded with Jacob’s wish regarding anonymous HIV testing. Undergoing anonymous HIV testing would have eliminated the dossier that could, in his opinion, be used by the local public health department to undertake law enforcement activities.

## Discussion

The quantitative component of this study revealed no statistically significant decline or increase in the number of HIV tests or diagnoses among MSM after the media releases in question. These findings suggest that, after the media releases of interest, at the population level, HIV testing continued without significant change among MSM, who are persons in the second group of our population health HIV prevention framework; i.e., members of a sexual networks with high HIV incidence and prevalence.^d^ See Figure 1. As Dodds [9] noted, however, significant changes in patterns of HIV testing may be unlikely because of the varying and conflicting influences that affect persons’ decisions to undergo HIV testing. Observing no changes should, accordingly, have been an expected outcome.

Moreover, this lack of statistically significant change in HIV testing and HIV diagnosis numbers should not have been surprising considering recent data on nondisclosure prosecutions. For one, Adam [21] reported that the majority of his Toronto-based participants supported nondisclosure prosecutions, thus highlighting that many persons’ HIV testing practices might not be affected by these prosecutions. Likewise, research from the UK generated similar results: Dodds [22] reported that, in a survey of 8252 gay men, the majority of participants (n = 4667 or 56.5%) felt that prosecutions were appropriate in situations of HIV transmission when HIV-positive serostatus disclosure had not occurred. More specifically, O’Byrne and colleagues [5], in their preliminary analysis of an Ottawa-based research that involved a convenience sample of 441 participants who identified as MSM, found that most respondents neither felt that “nondisclosure prosecutions made them afraid to talk to nurses or physicians about their sexual practices”, nor that these prosecutions “affected their decisions to undergo HIV testing”. In combination, these studies suggested, as was observed in our study, that one might not detect a population-level change in HIV testing and HIV diagnoses as a result of nondisclosure prosecutions or related media releases.

Burris and colleagues’ [8] work about the behavioural effects of nondisclosure prosecutions further supported this conclusion. In their survey of 248 persons who lived in Chicago and 242 persons who lived in New York City, these authors found that criminal laws which “regulat[e] sexual behaviour of HIV-infected” persons do not appear to affect most people’s sexual practices (p468). Similarly, Galletly and colleagues’ work [23], which involved statistical analyses of anonymous survey responses from a convenience sample of 479 persons who live in New Jersey, corroborated the idea that the criminal law has little impact on sexual behaviour. Whether or not Burris and colleagues’ [8] and Galletly and colleagues’ [23] findings apply to both HIV testing and the situation in Canada, however, remains unclear.^e^

Furthermore, in light of our above-noted findings, two other aspects of O’Byrne and colleagues’ [5] research are noteworthy. First, in O’Byrne and colleagues’ [5] study, among participants who were HIV-negative or unsure of their serostatus, those who noted that nondisclosure prosecutions either (a) affected their decisions to undergo testing or (b) made them reluctant to speak with nurses or physicians were more likely to have noted that they engaged in unprotected penetrative anal intercourse (*x*^2^, 5.47 (1), p = 0.019). Second, among the same group (HIV-negative or unsure of their serostatus), more respondents who noted they had never been tested for HIV indicated that nondisclosure prosecutions affected their testing practices (*x*^2^, 12.19 (1), p < 0.001) [5].

Because our findings are based on people’s actions, and not their self-reported behaviour, our population-level testing numbers raise interesting questions about O’Byrne and others’ [5] survey data. For one, the changes in our population-level HIV testing and diagnosis numbers might not have been significant because the persons who reported that these prosecutions affected their testing practices were so few in O'Byrne and colleagues' [5] study that they, therefore, would have had no impact on the population-level figures. In such a case, even if changes in testing occurred among a subset of MSM engaging in more unprotected sex and less frequent HIV testing, these changes may not have been noticeable at the aggregate-level. Alternatively, it could be that the persons who might were most influenced by nondisclosure prosecutions had previously decided not to be tested for HIV, thus indicating that such prosecutions are simply one additional reason for not undergoing HIV testing by persons already not accessing testing services As part of this, one must remember that, while the media releases of interest herein constituted what we believe are the first of such media releases in the local context, nondisclosure prosecutions from other Canadian regions have been occurring and have been publicized since the early 1990s. In this case, persons already not undergoing HIV testing due to nondisclosure prosecutions would not have induced any changes in our data. In addition, it is equally possible, in relation to nondisclosure prosecutions and HIV testing, that self-reported and actual behaviour do not align. In such case, what people say they will do and what they actually do may be different. It may simply be, furthermore, that people’s *post hoc* rationales differ from their initial, or at least originally stated, motivations. In any case, such possibilities warrant further investigation, which should elicit the information to either substantiate or refute the Supreme Court of Canada’s claims that nondisclosure prosecutions do not affect HIV testing practices.

While the quantitative component of our study did not point to any significant relationship(s) between nondisclosure prosecutions and public health HIV prevention efforts, our qualitative findings highlighted that nondisclosure prosecutions may have detrimental effects on both PHAs and prevention. Specifically, our data suggested that such criminal proceedings could negatively affect people aware they are HIV-positive, and could potentially undermine their HIV prevention abilities. This assertion arose from the qualitative finding that nondisclosure prosecutions have made some PHAs less willingly to both seek assistance from, and speak candidly with, public health workers about how to diminish onward HIV transmission. According to participants in this study, the reluctance to seek out and speak with such health professionals related to their belief that the local public health department shares information with police about instances of serostatus nondisclosure without requiring production notices, e.g., warrants.

When we considered this finding using the population health HIV prevention framework in Figure 1, it raised important questions about the extent to which nondisclosure prosecutions undermine the abilities of local public health officials to work with PHAs who wish to discuss what one of our participants called the “issues” he has had with safer sex and HIV prevention. Our qualitative data, therefore, suggested that nondisclosure prosecutions and their related media releases likely undermine HIV prevention involving the second highest priority group within our HIV prevention framework; i.e., PHAs who know they are HIV-positive, and who engage in practices that transmit HIV. See Figure 1. This scenario is undesirable from an HIV prevention perspective because, while Marks and colleagues argued that persons unaware of being HIV-positive are likely implicated in most HIV transmission, persons aware of their serostatus—particularly when they are asking for assistance to prevent HIV transmission—constitute an important group for HIV prevention [12,13].

This finding that nondisclosure prosecutions may hinder health professionals’ abilities to provide accurate, open, and supportive care for PHAs aligns with previous research. It corroborates Mykhalovskiy’s findings about how, on the one hand, public health workers have begun to undertake HIV prevention counselling with PHAs with an “eye to the law”, and how, on the other hand, this approach has impaired the abilities of these health professionals to, first, discuss serostatus disclosure and, second, counsel PHAs about how to address the “challenges they may be facing disclosing their HIV-positive status” (p.672) [4]. Similarly, our findings correspond with the issues raised during O’Byrne and Gagnon’s [24] discussion groups with frontline health staff in Ottawa: Nurses perceived that nondisclosure prosecutions have negatively affected their therapeutic relationships with PHAs, and, consequently, have undermined their abilities to provide appropriate and timely nursing care for these patients.

Moreover, other indications from our HIV-positive participants that criminal prosecutions made them wish they had undergone anonymous HIV testing, which would have resulted in the local public health department not possessing an identifiable record of their HIV-positive serostatus, might align with the current reality in Ottawa^b^. Local HIV testing and diagnosis figures indicated that anonymous HIV testing has the highest positivity rate (1.1% for anonymous testing, versus 0.26% for nominal testing), thus suggesting that people may select anonymous HIV testing if they believe the test will be positive [19]. Such findings may, furthermore, align with Lowbury and Kinghorn’s [25] arguments that nondisclosure prosecutions constitute a “clear disincentive to testing” (p.666), with the outcome not being an outright refusal to undergo HIV testing, but rather, the selection of anonymous rather than nominal HIV testing. Before absolute conclusions can be drawn about anonymous HIV testing using our data, however, further research is required to more precisely discern the relationship(s) between nondisclosure prosecutions and this HIV testing method. While our study examined *if* testing and diagnoses changed, more specific research should explore if nondisclosure prosecutions affect *who* undergoes testing, *when* they do so, and by *which* means. The answers to these questions could both reconcile the seemingly paradoxical findings which emerged in this study, and explain the partial differences between this study and those which preceded it.

Lastly, our other qualitative finding that some participants described PHAs as sexually promiscuous latent criminals raised a few important points for consideration. First, our participants’ linkage of HIV and the criminal law cast light on a potentially new aspect of the contemporary understanding that surrounds HIV and persons living with this virus. While the association between HIV and sexuality has long been documented [18,25-28], there has yet to be empirical accounts about people assuming a natural association between HIV, the criminal law, and latent criminal tendencies of PHAs. More research is needed on this topic, particularly from a sociological perspective.

The second aspect of our finding, that some participants linked HIV and the criminal law was that this belief added a new dimension to current knowledge about HIV stigmatization. From an HIV prevention perspective, because the extant literature identified that HIV stigmatization compromises PHAs’ abilities to maintain safer sex efforts with serodiscordant partners, nondisclosure prosecutions may be an unwanted addition [25-29]. Indeed, it may be a new item that can exacerbate HIV transmission [25-29]. In addition to the stigmatization that is already associated with an infection that is primarily sexually transmitted, [17] in our research, PHAs now appeared to be considered latent criminals; our participants used language, such as, “dangerous” and “murderer” to label PHAs, and made these associations based on serostatus, not sexual practices or behaviour. Therefore, because HIV stigmatization often corresponds with an increased probability of HIV transmission, our findings raise important questions about whether our participants’ association of PHAs with the criminal law, i.e., assumptions regarding latent criminality, could undermine PHAs’ psychosocial wellbeing, and, in turn, exacerbate HIV transmission at the population level.

Our research results thus corresponded with Dodds’ findings [22], which suggested that a hurdle to contemporary HIV prevention is the “direct conflict between the attitude changes that are necessary … for men to avoid acquisition of HIV and the way that these same men use popular narratives about criminal prosecutions to support their view about the way the world *should* be” (original emphasis, p.513). Our participants’ perceptions that PHAs were dangerous criminals supported Dodds’ [22] assertions that some MSM prefer to “blame” PHAs for HIV transmission, rather than focus on their personal responsibilities in HIV prevention. As noted by Dodds [22], such perceptions likely hinder population health HIV prevention efforts. Further research must clarify the precise linkages between this potentially new aspect of HIV-related stigmatization, and persons’ actual HIV prevention efforts. In the meantime, however, a potentially problematic situation exists for PHAs and for HIV prevention.

### Study limitations

These conclusions must be interpreted in light of certain limitations. Results cannot be generalized to all groups vulnerable to HIV described in the population health HIV prevention framework because this study only analyzed qualitative and quantitative data for MSM. This study recruited interview participants at venues frequented by these men, resulting in self-selection of men more connected to the gay community. Thus, participants did not fully represent the opinions of all MSM in Ottawa. Additionally, an inclusion criterion for the qualitative component of this study was awareness of the media release, whereas the extent of awareness of the media release was not assessed among the MSM who were tested for HIV after the media release. Thus, causation cannot be attributed to the media release or nondisclosure because other factors may have affected testing volume. Furthermore, the population of MSM vulnerable to HIV acquisition is unknown and so the data cannot be contextualized with a denominator. The unadjusted numbers for MSM tests are subject to reporting on the laboratory requisition, while the adjusted numbers are subject to the methodology used by the LEP. The 2008-2010 average annual response rate for the LEP survey sent to physicians with newly diagnosed patients from Ottawa was 55%, resulting in 69% of tests with known risk factor information prior to adjustment (2011 data not yet available).

### Need for further research

More studies are needed to explore the effects of nondisclosure prosecutions on population level HIV prevention. Researchers must undertake larger quantitative cohort studies that examine fluctuations in testing patterns over time, and in relation to legal changes and the testers’ attitudes and behaviours regarding nondisclosure and sexual practices. Data should also be gathered about the actual practices of individuals who seek testing, rather than just about their exposure information. This would allow for discrimination between, to use public health terminology, the *low-risk* and *high-risk* testers within each surveillance population; indeed, not all MSM who undergo HIV testing are at *high-risk* for HIV. In combination, such data could inform both population level HIV prevention strategies and the current legal context. Moreover, further research is needed to determine why approximately half of persons tested for HIV in Ottawa have no identified risk factors on the laboratory requisition because this risk-factor information helps establish HIV prevention policy, programming, and practice within public health departments.

## Conclusion

In this research, we undertook a two-part descriptive study that, quantitatively, examined the HIV testing and diagnosis numbers among MSM from January 2008 through December 2011, with a particular focus on identifying if these figures changed before versus after media releases about a local nondisclosure prosecution; qualitatively, we interviewed 27 local MSM to develop an in-depth understanding about their attitudes and beliefs about HIV serostatus disclosure, nondisclosure prosecutions, and public health. The quantitative component of this research identified an overall increase in HIV testing over the four-year period, but not did not find any significant changes in HIV testing or diagnoses pre versus post the nondisclosure prosecution media releases. The qualitative data, by comparison, highlighted the previously undocumented perceptions, first, that PHAs are latent criminals, and, second, that local public health departments openly share health information about PHAs with the police.

When these findings are considered in light of our HIV prevention model—wherein population health HIV prevention outcomes are linked to the proportional involvement of identifiable groups in onward HIV transmission—these results can be interpreted to suggest that nondisclosure prosecutions likely undermine population level HIV prevention. Specifically, the lack of significant changes in the quantitative data, which highlighted that HIV testing and diagnoses neither increased nor decreased among persons whose sexual group has high HIV incidence and prevalence, must be considered in hand with the qualitative findings, which identified that nondisclosure prosecutions compromise the HIV prevention efforts of some persons aware they are HIV-positive. That is, the qualitative data highlighted that nondisclosure prosecutions appear to contribute both to a novel form of stigmatization against PHAs (latent criminality), while these legal proceedings also seem to enhance the reluctance of some HIV-diagnosed persons who engage in practices that transmit HIV to approach public health nurses and physicians to discuss how they can diminish onward HIV transmission.

The balance of these two sets of findings is that, when our results are taken as a whole, the lack of significant changes in HIV testing and diagnoses, which is an otherwise neutral outcome among a large number of persons who were mostly HIV-negative, is offset by the negative findings that were being experienced and reported by a much smaller subset of individuals who (a) were aware of being HIV-positive, and (b) reported they engage in practices that transmit HIV. Problematically, the foregoing instances do not constitute potential situations where HIV transmission *could* occur, but rather, are known scenarios of HIV exposure wherein participants who were HIV-diagnosed noted that HIV transmission *is likely* occurring.

Our conclusion is, therefore, that nondisclosure prosecutions threaten HIV prevention efforts because, while persons who were overwhelmingly HIV-negative continued to undergo HIV testing without much change (quantitative findings), some HIV-diagnosed persons noted they do not seek the assistance they need to stop onward HIV transmission because they do not feel safe speaking with public health professionals (qualitative findings). On the whole, therefore, while many persons whose sexual group is disproportionately burdened by HIV continued to access HIV testing services, a smaller group of persons who are more implicated in onward HIV transmission noted these prosecutions undermine their HIV prevention abilities. Such findings highlight the need for more systematic policy and population-level analyses of the relationships between, and impact of, nondisclosure prosecutions on the health of populations.

## Endnotes

^a^ Since the submission of this manuscript for publication, and its subsequent review, the Supreme Court of Canada reviewed two nondisclosure prosecutions. In these cases, for which the decisions were released on October 5th, 2012, the Supreme Court of Canada reinforced and updated. *R. v. Cuerrier*. The new rulings are *R. v. Mabior and R. v D.C.*

^b^ While treatment as prevention may induce beneficial HIV prevention outcomes, which is our exclusive focus herein, this strategy creates ethical issues around informed consent (for testing and treatment initiation), access to treatment, perceptions of PHAs, and the meaning of undetectable viral loads, to name a few items. It is important that these points are considered at the forefront of all deliberations about treatment as prevention.

^c^ In the local area, a person can undergo HIV testing using an alphanumeric identifier. This prevents any reporting of this person’s name to the local public health department. This testing modality is known as anonymous HIV testing.

^d^ It would be erroneous to use our quantitative findings to discuss HIV testing and diagnosis among MSM unaware they are HIV-positive. While the lack of statistically significant changes for HIV testing and diagnosis could indicate that the proportion of people unaware they are HIV-positive did not change, it could also be that this proportion of persons either increased or decreased depending on the rate of HIV transmission during the same time. Without any understanding of HIV transmission numbers during the same time period, it is inappropriate to speculate about the meaning of our data in relation to persons unaware they are HIV-positive; (i.e., priority group 1a of our population health HIV prevention framework). Accordingly, we have restricted our interpretation to persons in sexual networks with elevated HIV incidence and prevalence; (i.e., priority group 2 from the same framework).

^e^ It is important to note that Burris and colleagues [8] extended their conclusions beyond the data they collected. These authors suggested that the criminal law would not affect HIV testing practices based on their findings that the criminal law does not affect sexual behaviour. Although Burris and colleagues’ [8] assertion about HIV testing may be valid, this conclusion needs to be verified empirically.

## Abbreviations

PHA: Person living with HIV/AIDS; MSM: Men who have sex with men; HIV: Human immunodeficiency virus; NIR: No information reported; STI: Sexually transmitted infection(s); OACHA: Ontario Advisory Committee on HIV/AIDS; LEP: Laboratory enhancement program; ANOVA: Analysis of variance.

## Competing interests

The authors declare that they have no competing interests.

## Authors’ contributions

POB was involved in all phases of the research, from conceptualization, to methodological design, to data collection and analysis (both quantitative and qualitative), to manuscript preparation. JW was the lead for the quantitative analysis portion of the project. AB undertook all qualitative recruitment, data collection, and contributed to research design and manuscript preparation. DSF contributed research design, quantitative analysis, and manuscript preparation. AH contributed to research design, qualitative analysis, and manuscript preparation. CH contributed to research design, qualitative analysis, and manuscript preparation. DM contributed to research design, qualitative analysis, and manuscript preparation. CB contributed to qualitative recruitment / data analysis, and manuscript preparation. RSR contributed to quantitative analysis with LEP, and manuscript preparation. VE conceptualized and oversaw research, in addition to participating in the qualitative and quantitative analysis, and manuscript preparation. All authors read and approved the final manuscript.

## Pre-publication history

The pre-publication history for this paper can be accessed here:

http://www.biomedcentral.com/1471-2458/13/94/prepub

## owledgements

The authors would like to recognize that this project would not have been possible without funding from Ottawa Public Health (VE), the Ontario Government’s Early Researcher Award Program (POB), and the AIDS Bureau of Ontario (RSR). Juan Liu at the Ontario HIV Epidemiologic Monitoring Unit helped to carry out the adjustments in the LEP.
